# Antimicrobial use and antimicrobial resistance in *Escherichia coli* in semi-intensive and free-range poultry farms in Uganda

**DOI:** 10.1016/j.onehlt.2024.100762

**Published:** 2024-05-23

**Authors:** Irene Mbatidde, Dickson Ndoboli, Dreck Ayebare, Dishon Muloi, Kristina Roesel, Linnet Ochieng, Michel Dione, Bernd-Alois Tenhagen, Savino Biryomumaisho, Eddie Wampande, Barbara Wieland, John Elmerdahl Olsen, Arshnee Moodley

**Affiliations:** a International Livestock Research Institute, Kampala, Uganda; b International Livestock Research Institute, Nairobi, Kenya; c International Livestock Research Institute, Dakar, Senegal; d Federal Institute for Risk assessment, Berlin, Germany; e Department of Veterinary Pharmacy, Clinical and Comparative Medicine, Makerere University, Kampala, Uganda; f Institute of Virology and Immunology, Bern, Switzerland; g Department of Clinical Veterinary Science, University of Bern, Bern, Switzerland; h Department of Veterinary and Animal sciences, University of Copenhagen, Copenhagen, Denmark; i National Agricultural Research Organization, Mbarara, Uganda; j Institute of Infection, Veterinary and Ecological Sciences, University of Liverpool, Liverpool, United Kingdom

**Keywords:** Antibiotic, *Enterobacteriaceae*, Chicken, Stewardship, LMIC, Africa

## Abstract

Livestock associated antimicrobial resistance (AMR) can reduce productivity and cause economic losses, threatening the livelihoods of poor farming communities in low-income settings. We investigated the practices and risk factors for increased antibiotic use, and AMR in *Escherichia coli* including resistance to human critically important antibiotics like cefotaxime and colistin in semi-intensive and free-range poultry farms in Uganda. Samples and farm management data were collected from 402 poultry farms in two districts between October 2021 to March 2022. Samples were processed to isolate *E. coli* and to quantify cefotaxime (CTX) and colistin (COL) resistant coliforms*.* The identification of presumptive *E. coli* isolated on MacConkey agar without antibiotics, was confirmed by matrix-assisted laser desorption/ionization-time of flight mass spectrometry and subjected to antimicrobial susceptibility testing by disk diffusion using EUCAST guidelines. Our models indicated that antibiotic use was associated with production intensity, and type of feed used. Moreover, semi-intensive farmers had better knowledge on antibiotic use compared to farmers in the free-range system. In semi-intensive farms, 52% harbored COL^R^ and 57% CTX^R^ coliforms. In free-range farms, 54% had COL^R^ and 67% CTX^R^ coliforms. Resistance to tetracycline, ampicillin and enrofloxacin were more frequent in semi-intensive farms compared to the free-range farms. Multi-drug resistant *E. coli* were identified in both poultry production systems despite different management and antibiotic use practices. There was no significant relationship between antibiotic use and resistance for the six antibiotics tested.

## Highlights

• Antibiotic use was associated with production intensity and type of feed used.
• Semi-intensive poultry farmers had better levels of knowledge on antimicrobial use.
• Colistin and cefotaxime resistance was frequent in both production systems.

## 1 Background

Antimicrobial resistance (AMR) is one of the top ten global health challenges with low and middle-income countries (LMICs) disproportionately affected [1]. AMR in bacteria of livestock origin is rising particularly in LMICs with the largest increase observed in pigs and poultry [2]. Misuse and overuse of antimicrobials have been highlighted as major drivers for AMR [3].

Poultry production is one of the fastest growing livestock sectors in Uganda. Poultry numbers have increased from 32.5 million in 2008 to 43.1 million in 2021 [4], and the sector is important as it contributes to household income, food security, and the national economy [5]. Poultry in Uganda is raised in three different production systems: 1) intensive with permanent housing structures and rearing >1000 chickens 2) semi-intensive with semi-permanent to permanent structures chickens that are housed exclusively indoors and farms having 200–1000 birds, and 3) free range system with semi-permanent houses, where chicken are housed indoors only at night and keep 1–200 chickens. Most Ugandan farmers (55%) produce chickens in the free-range system followed by the semi-intensive system (25%) and intensive system (20%) [6].These systems are also associated with differing levels of antimicrobial use and biosecurity [7]. Previous studies have shown that intensive livestock production systems are more likely to have higher antibiotic use compared to the other production systems [8].Various antimicrobials are licensed for use in livestock, and these are listed in the Ugandan Essential Veterinary Medicines List 2020 [9], and according to the legislation, antibiotics can only be used under the supervision of registered veterinary personnel [9]. A recent study among pig and poultry producers in Wakiso Uganda, showed that antibiotics were commonly used prophylactically and for growth promotion, and was largely associated with protection of livelihoods [10].

On the other hand, AMR has also been reported in livestock in Uganda. Of 134 *E. coli* isolates from commercial livestock farms in Wakiso and Mpigi districts, 97% were resistant to tetracycline, 56.7% to sulfamethoxazole-trimethoprim, and 44.8% to ampicillin [11]. In another study on *E. coli* from broiler chickens in central and northern Uganda, resistance to tetracycline, ampicillin, trimethoprim-sulfamethoxazole, and chloramphenicol were reported [12]. This study aimed to compare antimicrobial use (AMU) practices in semi-intensive and free-range systems, which accounts for 75% of poultry produced in Uganda, as well as measuring AMR in the two production systems.

## 2 Materials and methods

### 2.1 Study area and ethical clearance

The study was conducted in Uganda in two districts: Wakiso and Soroti. Wakiso is in central Uganda surrounding Kampala city. It has a total area of 2807.75 km^2^ with 1,997,418 people [13]. Wakiso has the largest number of poultry in the country, approximately 2,783,509 chickens [13], with semi-intensive (i.e. chickens that are housed exclusively indoors and farms having 200–1000 birds) to intensive (i.e. farms having >1000 chickens) farms. The chicken breeds reared are predominantly exotic breeds e.g. leghorn, Marans, and Cornish Cross but can also include, to a lesser extent, indigenous breeds. Soroti is located in eastern Uganda (approximately 280 km from Kampala) covering an area of 2662.5 km^2^with a total population of 296,833 people [13]. Soroti has a chicken population estimated at 808,290 chickens [14], and production is characterized by free-range farms (i.e. chickens housed indoors at night only), having 1–200 birds, and mainly comprising of indigenous breeds used for dual-purposes (i.e. egg laying and chicken meat). The two districts were selected because of their differing production systems and landscapes i.e., *peri* urban Wakiso and rural Soroti.

### 2.2 Study design, sampling, and farm selection

We used a cross-sectional study design on 402 farms that were randomly selected from the two districts and production system types: 200 semi-intensive farms and 202 free range farms. A sample size of 180 farms per production system was calculated to allow a detection of 0.13 difference in antimicrobial use per production system if one of the proportions of use in one was 0.1 and the other was 0.23 (https://epitools.ausvet.com.au/twoproportions). The power need was based on studies by [8] which highlight such a difference in AMU among semi-intensive and free-range systems. The sample size was adjusted to 200 farms per population to cater for non-responsiveness.

### 2.3 Questionnaire

A modified version of the ‘Antimicrobial use in livestock production systems’ questionnaire (AMUSE Livestock tool) developed by the International Livestock Research Institute (ILRI) [15] was used to collect data regarding farmers' knowledge and practices. The questionnaire was previously used in Ethiopia [16], Uganda [17] and Burkina Faso [18]. It comprises of 75 questions covering: 1) farm and household demographics, 2) farm characteristics, 3) management of manure, feed, and water, 4) animal health and disease prevention, 5) utilization of animal health services, 6) veterinary drug use including a specific focus on antibiotics. Written informed consent was obtained from either the owner/designated farm worker and were interviewed by a field researcher and the local government extension officer who also acted at times, as a translator, translating the questions and answers in the local language. Each farm had a unique identifier, and all responses were uploaded via the Open data Kit (ODK) software and stored on a secured ILRI data server. The full questionnaire is provided in the supplementary material (Supplementary table S1).

### 2.4 Sample collection

A composite faecal sample and a boot sock sample were collected from one chicken house on each farm. For the composite sample, 10 g of freshly voided chicken droppings was collected using a hand glove and kept in a sterile sample collection bottle. The sample of boot socks was collected according to the European Commission regulation No 200/2012 for detection of *Salmonella* in poultry flocks. A boot sock consisting of 10 cm of tubular gauze (ConvaTec Tubigrip, size D, 3 in.) was worn over a plastic covered boot. The researcher then walked inside the poultry house, first longitudinally, then transversally. This was to ensure that all sections in the poultry house were sampled. The boot sock was carefully removed from the boot as not to dislodge adherent material, inverted, then placed in a sterile Ziplock bag and labelled with the unique farm identification number. All samples were kept at 4 °C and transported to the laboratory for processing within 24 h.

### 2.5 Quantification of colistin and cefotaxime resistant coliforms

For quantification of cefotaxime (CTX^R)^ and colistin (COL^R^) coliforms*,* 1 g of the composite sample was added to 9 ml buffered peptone water, which were homogenized, and 10-fold serially diluted in saline (10^−1^ -10^−6^). One hundred μl of each dilution was spread onto MacConkey agar plates (Oxoid) supplemented with cefotaxime (final concentration 3 μg/ml) and colistin (final concentration 3 μg/ml), and antibiotic-free MacConkey, and incubated aerobically overnight. The concentration chosen are above the epidemiological cut off values for each antibiotic (CTX = 0.25 μg/ml and COL = 2 μg/ml, (https://www.eucast.org/mic_and_zone_distributions_and_ecoffs) After overnight incubation, colonies were counted on each dilution and the colony-forming units (CFU/g) was calculated. A single colony from each plate (antibiotic containing and antibiotic free), was further sub-cultured on 5% blood agar and stored at -20 °C for further analyses.

### 2.6 Confirmation of bacterial species identification and antimicrobial susceptibility testing of *E. coli* isolated on antibiotic free MacConkey

All presumptive *E. coli* isolated from MacConkey containing not antibiotics, was revived on Nutrient agar and identified on the MALDI Biotyper Smart System IVD (Bruker Daltonik). The direct spotting method was used following the manufacturer's instructions i.e. a small amount of bacterial mass from one isolated colony was transferred onto the MALDI-TOF steel target plate using a sterile toothpick, coated with 1 μl of HCCA matrix, allowed to dry at room temperature and subjected to MALDI-TOF MS analysis using the MBT Compass reference library. Antimicrobial susceptibility test (AST) was performed according to EUCAST guidelines. Between 3 and 5 colonies were resuspended in saline to obtain a 0.5 McFarland turbidity using a DensiCheck (Biomerieux). A sterile swab was dipped into the bacterial suspension and evenly spread on a Mueller Hinton agar plate using a C80 Rota plate inoculator. Antibiotic discs were then applied within 15 min of inoculation using a dispenser and plates were inverted and incubated aerobically at 35 °C for 18 ± 2 h. *E. coli* ATCC25922 was included as a QC reference strain. The following antibiotics were tested: ampicillin, amoxicillin-clavulanic acid, ceftiofur, trimethoprim-sulfamethoxazole, tetracycline, cefotaxime, enrofloxacin, gentamicin. These are among the commonly used antibiotics on poultry farms. Inhibition zones diameters were interpreted using the EUCAST breakpoint tables.

### 2.7 Statistical analysis

The data were analyzed in Stata/SE 17.0 and in R version 4.2.3. Descriptive statistics summarizing the different percentages of farmers and their practices in both the semi-intensive and the free-range system were generated. To investigate farm-level risk factors for antibiotic use, the “number of times” each farm used antibiotics in the last 4 weeks before the interview was used as the outcome variable. Zero inflated models were used because the data was over dispersed with a high number of zeros in the response variable tested. Both univariate analysis and multivariate analysis were used to test the association of risk factors with the outcome. Model explanatory variables included: farm type, number of birds kept, housing, chicken type kept (layers, broilers, dual purpose), feed source, having a foot bath, disinfecting the premises before every stocking, distance from agrovet shop, having been visited by a drug company representative, use of vaccines, source of veterinary drugs, source of veterinary advice, reported a disease in the previous month, keeping other farm animals and keeping other avian species.

## 3 Results

### 3.1 Farmer and farm demographic characteristics

In semi-intensive farms in Wakiso, 54% of respondents were female and in the free- range farms in Soroti, 46% of respondents were female. Most respondents were between 18 and 49 years old (Table 1). Most farms (43%) in the semi-intensive production kept between 200 and 1000 chickens, and 27% had over 1000 chickens. In the free-range system, farmers kept between 1 and 200 chickens (97% of farms). There was a difference in the chicken type or rearing purpose in the two systems. Semi-intensive farms had a combination of egg-layers (38%), broilers (33%) and dual purpose (combined egg-laying and broiler breed, 29%), while almost exclusively, farms in free-range system had dual purpose breeds (99.5%).

**Table 1:** Farm demographics and on-farm biosecurity practices in semi-intensive and free-range poultry farms in Uganda.

Variable	Category	Semi-intensive (%), *n* = 200	Free-range (%), *n* = 202
Age group			
	18–49 years	154 (77%)	129 (64%)
	50–69 years	44 (22%)	63 (31%)
	> 70 years	2 (1%)	10 (5%)
Gender			
	Male	92 (46%)	109 (54%)
	Female	108 (54%)	93 (46%)
Education level			
	Primary	114 (30%)	140 (69%)
	Secondary	76 (38%)	34 (17%)
	Tertiary	64 (32%)	28 (14%)
Flock size			
	1–200	60 (30%)	196 (97%)
	201–1000	86 (43%)	4 (2%)
	>1000	54 (27%)	2 (1%)
Chicken type kept			
	Broilers	66 (33%)	2 (1%)
	Layers	76 (38%)	0
	Dual Purpose	58(29%)	200 (99%)
Management of dead chickens			
	Eat	8 (4%)	82 (41%)
	Bury	64 (32%)	44 (22%)
	Burn	16 (8%)	3 (1.4%)
	Dispose in rubbish pits	58 (29%)	73(36%)
	Feed to other farm animals	54 (27%)	0
Disinfection at the farm			
	Foot bath present-Yes	60 (30%)	2 (1%)
	If present, contained disinfectant-Yes	54 (27%)	2 (1%)
Practices to keep birds healthy			
	Cleaning houses	76 (38%)	65 (32%)
	Used vet drugs	38 (19%)	87 (43%)
	Good feeding⁎	30 (15%)	36 (18%)
	Using supplements	16 (8%)	10 (5%)
	Fence around the farm	12 (6%)	2 (1%)
	No mixing with chickens from other farms	28 (14%)	2 (1%)
	Use of vaccines	188 (94%)	69 (34%)

⁎ Good feeding related to providing chicken with a nutritional and well-balanced diet.

### 3.2 Biosecurity measures on the farms

Farms in both production systems had varying levels of biosecurity (Table 1). Semi-intensive farms in Wakiso had more biosecurity measures than free-range system in Soroti. In the semi-intensive farms, one third of them had footbaths at the poultry house entrance compared to 1% of free-range farms in Soroti. Likewise, 27% of farmers in the semi-intensive system reported using disinfectants in the footbaths compared to 1% of farmers in the free-range system. Biosecurity measures for maintaining flock health differed between production systems, with regular cleaning of chicken houses considered effective by <40% of farmers in both systems. Vaccine use was higher in semi-intensive farms (90%) compared to free-range farms (34%), while 43% of free-range farmers relied on veterinary drugs for maintaining chicken health.

### 3.3 Knowledge of antibiotic use by farmers

The level of knowledge about rational antibiotic use was assessed by asking farmers three questions. Good knowledge was equated to answering all three questions correctly i.e.: yes, no, I don't know. We asked farmers “Should antibiotics be used to treat sick chickens?”, 96% vs 63% answered correctly in the semi-intensive and free-range, respectively. When asked “Should antibiotics be used to prevent disease in chickens?”, 48% vs. 49% got the answer wrong in semi-intensive and free-range farms, respectively. When asked “Should antibiotics be used to fatten chickens?”, 48% vs.37% got it wrong in semi-intensive system and free-range, respectively. According to our categorization, only 24 respondents (12%) in the semi-intensive system and just three respondents (1.5%) in the free-range had good knowledge of rational use of antibiotics. (Supplementary Figs. S1 and S2).

### 3.4 Antimicrobial use practices

**Fig. 1:**
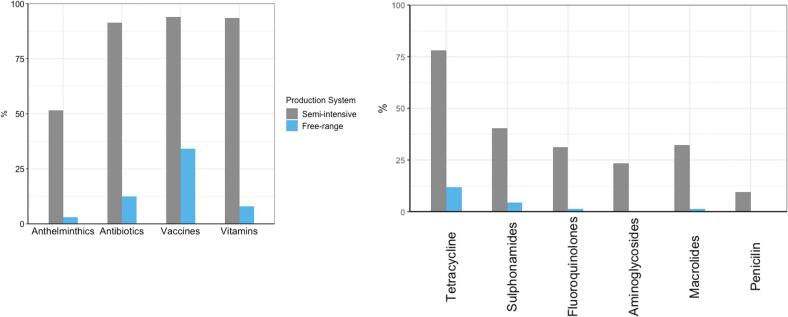
A) Percentage of farms that reported using different veterinary medicines, and B) antibiotics, in the previous four weeks in semi-intensive and free-range poultry production systems in Wakiso and Soroti, respectively.

Farms in both production systems reported using a variety of veterinary medicines including antibiotics, anthelmintics and multi-vitamins, with significantly higher usage in semi-intensive farms compared to the free-range system (*p* = 0.001, chi square test, Fig. 1a). There was a notable difference in antibiotic use between the two farming systems, with 95% of semi-intensive farms in Wakiso reporting recent antibiotic usage compared to 54% of free-range farms in Soroti. Tetracycline was the most used antibiotic in both farming systems, with higher usage reported in the semi-intensive system in Wakiso (78%) compared to free range system in Soroti (12%), followed by sulfonamides, macrolides, and fluoroquinolones (Fig. 1b). Farmers reported frequently using antibiotic and multivitamin combinations in the semi-intensive system compared to the free-range system. These combinations contained one or more antibiotics, namely oxytetracycline, neomycin, colistin, erythromycin and streptomycin.

Of the surveyed farms, 73% in Wakiso and 98% in Soroti reported using antibiotics for therapeutic reasons, while 42% in Wakiso and 8% in Soroti used antibiotics for prophylactic purposes. Additionally, the use of antibiotics for growth promotion was reported in 8% of farms in the semi-intensive system and 4% in the free-range system. Cough and diarrhea were the commonly reported signs for antibiotic use in both systems, with higher frequencies observed in the free-range system (73% and 60%, respectively) compared to the semi-intensive system (55% and 26%, respectively). Antibiotics were frequently administered contrary to the manufacturers' recommendations, and by some farmers for up to 30 days continuously instead of the recommended 3–7 days (Table 2). Most farmers (95.5%) in Wakiso reported acquiring antibiotics from veterinary drug shops or agrovets, while 39% of farmers in Soroti relied on traditional medicine like herbal remedies.

**Table 2:** Frequency of antibiotic use in the preceding four weeks prior to the farm visit in semi-intensive and free-range poultry production systems in Wakiso and Soroti, respectively.

Antibiotic	Number of treatment days recommended by the manufacturer	Production System	No. of farmers reported use longer than the recommended period	Most number of days the antibiotic was used
Tetracyclines	7	Semi-intensive	22	30
		Free- range	0	5
Penicillin	3–5	Semi-intensive	3	14
		Free-range	0	14
Aminoglycosides	3–5	Semi-intensive	3	30
		Free-range	0	0
Fluoroquinolones	3–5	Semi-Intensive	12	30
		Free-range	0	2
Sulphonamides	3–5	Semi-intensive	8	10
		Free-range	0	5
Macrolides	3–5	Semi-intensive	5	10
		Free-range	0	2

We observed on farms the use of medicines designated for humans to treat conditions like malaria, pain, fever, cough, and bacterial infections that were being used in poultry when chickens showed signs of infections like flu, coughing, and malaise. This was mostly observed in the free-range system (22% of farms) compared to the semi-intensive system (14% of farms). The commonly used human medicines included Panadol (paracetamol), Coartem (artemether/lumefantrine), Ampiclox (ampicillin), AB-DOX-100 (doxycycline), Cloxapen (cloxacillin), and Septrin (trimethoprim/sulfamethoxazole), which are readily available over the counter at pharmacies, local community health offices, and village health clinics.

### 3.5 Factors influencing antibiotic use on farms

Our univariable analysis revealed significant associations between antibiotic use and several putative drivers (Table 3). The final multivariable model revealed significant associations between a higher frequency of antibiotic use and farms that reported disease in the preceding three months (OR = 1.33, *p* = 0.05, 95% CI [1.00–1.77], larger farm sizes (201–1000 chickens: OR = 2.57, *p* = 0.001, 95% CI [1.67–3.96]; >1000 chickens: OR = 4.53, p = 0.001, 95% CI [2.83–7.25]), and the utilization of commercial feed (OR = 9.74, p = 0.001, 95% CI [6.26–15.15]).

**Table 3:** Univariable analysis for different predictor factors.

Variable	Estimate	Standard Error	Z value	Pr(>(z)
Drug source official (ref. nonofficial source)	1.57	0.4	4.02	5.76 *e*-5
Source of advice profession (ref. peer advice)	1.07	0.3	3.65	2.6 *e*-4
Use lab services (ref don't use)	0.03	0.17	0.18	0.86
Received disease Prevention training (ref. did not receive)	−0.12	0.14	−0.83	0.41
Reported disease (ref. not reported disease)	0.31	0.16	1.926	0.05
Keep farm animals (ref. not keeping farm animals)	−0.25	0.14	−1.78	0.083
Keep other avian species (ref. not keeping other avian species)	−0.79	0.19	−4.16	3.19 *e*-5
Disinfect premises before rearing (ref. not disinfecting)	2.98	0.16	18.7	2.0 *e*-16
Commercial feeds (ref. conventional feeds)	3.29	0.17	19.24	2.0 *e*-6
Had a pharma visit (ref.no pharma visit)	−0.12	0.18	−0.68	0.49
Keep 201-100chicken (ref. keep1-200chicken)	2.38	0.17	13.41	2.0 *e*-16
Keep over 1000chicken (ref. keep 1-200chicken	3.12	0.2	15.5	2.0 *e*-16
Chicken type layers (ref. dual purpose)	1.62	0.22	7.31	2.5 *e*-15
Chicken type broilers (ref. dual purse)	1.76	0.25	7.13	9.6 *e*-13
Chicken type mixed (ref. dual purpose)	1.87	0.29	6.33	2.3 *e*-10
Housing fully indoors (ref. outdoors)	2.47	0.15	16.10	2.0 *e*-16

Manuscript figures including figure legends; Yes color should be used for these figures.

### 3.6 Quantification of cefotaxime (CTX^R)^ and colistin (COL^R^) resistant coliforms on MacConkey agar supplemented with antibiotics and without antibiotics

**Fig. 2:**
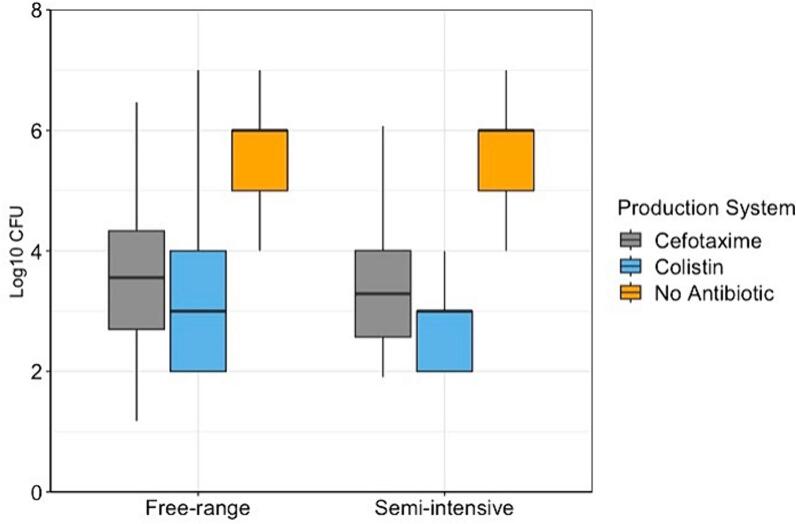
Log10 CFU distributions from plates with cefotaxime, colistin and plates without antibiotics from semi-intensive and free-range poultry farms in Uganda.

Fig. 2 shows the box plots of the Log10 CFU counts from the composite faecal samples isolated on MacConkey agar plates with and without antibiotics in semi-intensive and free-range farms. No significant difference was observed between the CFU counts in the two poultry systems.

### 3.7 Antimicrobial susceptibility testing on *E. coli* isolated on antibiotic-free MacConkey agar

**Fig. 3:**
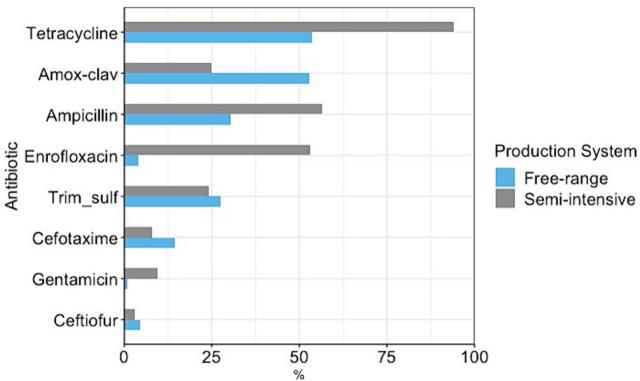
Antibiotic resistance among 312 *E. coli* isolated on antibiotic-free agar against eight antibiotics from semi-intensive and free-range poultry farms in Uganda.

Of the 355 samples that grew presumptive *E. coli*, 312 isolates were confirmed by MALDI-TOF MS and further tested for susceptibility to a panel of eight antibiotics (Fig. 3). >90% of isolates from semi-intensive farms were resistant to tetracycline and > 50% were resistant to ampicillin and enrofloxacin (using the ciprofloxacin ECOFF = 25 mm). Whereas in free-range farms, 53% of isolates were resistant to tetracycline and amoxycillin-clavulanic acid but limited resistance to fluroquinolones was observed in *E. coli* from free range farms. Forty-seven isolates (15%) were fully susceptible to all tested antibiotics, while 44% of isolates (*n* = 138) were resistant to ≥3 or more antibiotic classes.

## 4 Discussion

This study aimed to investigate whether there were differences in AMU practices between semi-intensive and free-range poultry farms in two districts in Uganda, and whether this could be reflected in the occurrence of antimicrobial resistant *E. coli*. Several differences in practices were observed between the two production systems. These include use of vaccination, the sourcing of medicine from agrovets, keeping records of antimicrobials administered to chickens, utilization of diagnostic laboratory services and veterinary services, and farmers having received training on disease prevention. These practices were more common in semi-intensive farms where the number of chickens were greater compared to the free-range farms which is generally typified by smaller flock sizes (<200 chickens). Similar observations were seen in Vietnamese poultry farms suggesting that as farmers progress to larger, more intensive farming, this is complemented with better animal husbandry practices [19].

Our analyses indicated that farms with higher numbers of chicken used more antibiotics compared to those with lesser numbers of chicken. This frequent use may be attributed to the larger flock size that are kept in close confinement, which is a predisposing factor for infection [20]. But it may also reflect that these larger farms have invested more financial resources and are able to afford antibiotics, and may be driven to use antibiotics to protect their investments and their livelihoods [10]. To further support this, most farmers in the free-range, small-scale farms in rural Soroti reported using more traditional medicines rather than antibiotics. A similar observation made by Ndukui et al. who found that intensive poultry producers in Kenya used more antibiotics than less intensive systems [21] and that the average number of birds on a farm influenced both the level of knowledge and practice on antibiotic use.

We also observed that farms that used commercial feeds were ten times more likely to use antibiotics compared to farms that used conventional feeds (i.e., house-hold waste and scavenging). This could be due to farmers being able to access other farm inputs including antibiotics when they purchase these feeds. They are also more likely to get advice from the feeds company or feeds sellers on which antibiotics to use, likely driving their frequent use of antibiotics. Our findings agree with those of Al Masud et al. in Bangladesh, who found that poultry farmers received credit and advice from poultry dealers, these farmers in turn were obliged to buy poultry feeds and medicine from these dealers [28].

Farmers in the semi-intensive systems not only had higher AMU but also reported using multi-vitamins and vaccines more frequently, which could be seen as good animal husbandry practices that should be related to reduced dependency on antibiotic use as vaccines prevent diseases and vitamins boost immunity and improve appetite. However, upon further examination of the ingredients of the multi-vitamins packages we found on farms, they contained in addition to vitamins, between 3 and 5 different antibiotics such as oxytetracycline, neomycin, colistin, erythromycin and streptomycin. These products are registered as multi-vitamins in Uganda, cost approximately $6 per 100 g package recommended as supplements for chicken with poor condition, decreased appetite, or stress. Hence multi-vitamins are well favored by poultry farmers. This observation agrees with Jibril et al. who found that most vitamins in Nigeria were mixture of vitamins and antibiotics [22]. Similarly in study in Kenya by Kiambi et al., found vitamin mixed with antibiotics were commonly used to boost egg and meat production as well as preventing stress [23]. It should be noted that this antibiotic containing multi-vitamins used to promote growth has been banned in the European Union since 2005, and since these products are not registered as containing antibiotics, they are excluded from reporting to WOAH as part of the global AMU surveillance. There is a possibility that farmers are not aware that these multi-vitamins contain antibiotics, and points to the sources of advice farmers use regarding AMU.

Our study showed that only 40% of farmers seek animal health advice from qualified veterinary practitioners while the remaining farmers obtain their AMU advice from community animal health workers, drug shops attendants, friends/neighbors or reading the drug labels themselves. Our results are similar to Kariuki et al 2023, who reported that Kenyan farmers obtained AMU information from drug sellers, suppliers of day old chicks and fellow farmers [24]. Lastly, farmers' level of knowledge on antibiotic use was low (1.5% vs 12%) for free-range and semi-intensive system, respectively. These findings are consistent with a recent systematic review that highlighted knowledge and practice gaps among poultry farmers regarding antimicrobial use. [25]

We observed that the most used antibiotics were tetracyclines, sulphonamides, fluoroquinolones, and macrolides. These antibiotics are commonly used in Africa as reported in the 6th annual report on antimicrobials intended for animals [26] with tetracycline making 53% of the total proportion of antibiotics consumed. It was noted that the dosage regiments as recommended by the manufacturer were not adhered to by the farmers. It is unclear if this is because farmers were not aware of how to use the antibiotic properly or were not instructed adequately on how to administer the antibiotic. For example, farmers reported using tetracyclines and fluroquinolones for up to 30 days continuously, which is only meant to be used 3–5 days for treatment. The purpose of this long-term, sustained use was for both disease prevention and growth promotion. This continuous administration of antibiotics is irrational and a major driver of AMR [3]. Our results agree with similar observations in poultry farms in Burkina Faso and in Ethiopia who reported that up to 72% of farmers did not follow the recommended treatment course while treating their animals and generally lacked knowledge on the rational use of antibiotics [18] [16]. Moreover, the most frequently used antibiotics in poultry belong to either the “Watch” or “Access” groups, and aside from colistin, there appears to be little use of other highly critically important antibiotics for human health, which compare well with results found in other studies in LMICs [8,16,21,27].

>50% of our *E. coli* isolates were resistant to tetracycline, ampicillin, amoxicillin-clavulanic acid and enrofloxacin. This is also reflected by the use of these antibiotics on farms. *E. coli* from the free-range system were more resistant to tetracycline, trimethoprim-sulfonamide, and amoxicillin -clavulanic acid. Whereas isolates from semi-intensive farms were more resistant to tetracycline, enrofloxacin and ampicillin. We observed more resistance to the human critically important antibiotics like cefotaxime and amoxicillin-clavulanic acid in the free-range system. This result may be associated with the use of human drugs in this area. For example, amoxicillin-clavulanic acid that was designated for human use, in Soroti's free-range farms, this preparation was used to treat chickens. This use of amoxicillin-clavulanic acid is largely related to the availability and access of this antibiotic formulation from human health care facilities. The practice of cross over use has been noted by other studies in Uganda like Ndoboli et al 2019 who found the use of antiretroviral drugs to treat infections and boost pig and poultry productivity [17].

We observed a high prevalence of CTX^R^ in both production systems even though 3rd generation cephalosporins are not registered for use in poultry in Uganda, and we did not find them on farms. Similarly, we found COL^R^ coliforms in both systems. While colistin is registered for use in poultry, we did not find colistin on farms except in formulations combined with multi-vitamins that were routinely used. The inclusion of this “last resort” antibiotic in poultry multi-vitamins used for growth promotion is worrying and should be targeted for a ban to preserve their effectiveness in humans. Studies in China and other countries where colistin is banned from use in livestock, have reported a significant reduction in the isolation of *Enterobacteriaceae* resistant to colistin [29,30].

There were some limitations in this study for example our approach to quantify AMU was based on memory recall of what drugs farmers used in the four weeks preceding our visit. Moreover, in the local languages spoken in Wakiso and Soroti, there is not a specific word for antibiotics and antibiotics are typically lumped together and called generically medicines. These two limitations hamper attempts to quantify AMU in the absence of any record keeping/surveillance. Despite these limitations, our study showed differences in AMU practices within the two production systems and varying levels of AMR. Collectively, our findings suggest the need for increased knowledge and awareness on the rational use of antibiotics and a ban or reclassification of multi-vitamins containing antibiotics.

## 5 Conclusion

The study established different AMU practices and amounts of antimicrobials used in two different farming systems in Uganda. The major drivers for AMU were associated with production intensity and types of feed used. Multi-drug resistant *E. coli* were identified in both poultry production systems despite different management and AMU practices including resistance to critically important human drugs, colistin and cefotaxime. There was no significant relationship between antibiotic use and resistance for the six antibiotics tested. These findings highlight 1) the need for educational and antimicrobial stewardship programs tailored to the specific needs of semi-intensive and free-range farming systems and 2) the need for improved regulations that control the use of human critically important antibiotics, such as cefotaxime and colistin, in agriculture.

## Ethics statement

Permission to conduct the study was granted by the Ugandan National Council for Science and Technology (reference number: A166ES), from Makerere University, School of Veterinary Medicine Animal Resources (SVAR), and the Institutional Animal Care and Use Committee (IACUC) (reference number: SVAR_IACUC/77/2021) and the Institutional Research Ethics Committee (IREC2021–59) at the International Livestock Research Institute. Written informed consent was obtained from all the participants.

## Funding

This work was funded by the German Federal Ministry of Economic Cooperation and Development (BMZ) through the project Boosting Uganda's Investment in Livestock Development (BUILD). Additional support was received from the CGIAR Research Programs on Livestock and Agriculture for Nutrition and Health (A4NH).

## CRediT authorship contribution statement

Irene Mbatidde, Dickson Ndoboli and Dreck Ayebare: Conceptualization, Formal Analysis, Methodology, Investigation, Writing. Kristina Roesel: Conceptualization, Writing, Funding acquisition. Michel Dione, Linnet Ochieng, Dishon Muloi: Conceptualization, Writing. Bernd-Alois Tenhagen, John E Olsen: Conceptualization, Supervision, Writing. Barbara Weiland: Conceptualization, Writing. Arshnee Moodley: Conceptualization, Funding acquisition, Methodology, Supervision, Writing.

## Declaration of competing interest

We confirm that there are no known conflicts of interest associated with this publication and there has been no significant financial support that could have influenced its outcome. We confirm that the manuscript has been read and approved by all authors and that the order of authors listed in the manuscript has been approved by all.

## Data Availability

The data referenced in this study is retrievable upon request from the corresponding author and supplementary material is submitted herein. All isolates from the study are in the data repository in the Ministry of Agriculture Animal Industry and Fisheries MAAIF and can be availed through request from the project.

## Appendix A Supplementary data

Figures and AMUSE questionnaire Image 1
