# Supplementary material for: Antimicrobial use and antimicrobial resistance in Escherichia coli in semi-intensive and free-range poultry farms in Uganda
Source: One Health. 2024 May 23;18:100762. doi: 10.1016/j.onehlt.2024.100762 (PMC11190498; doi:10.1016/j.onehlt.2024.100762)
Supplement: Supplementary file 1 — Figures and AMUSE questionnaire [file mmc1.docx]

**Supplementary Figure**


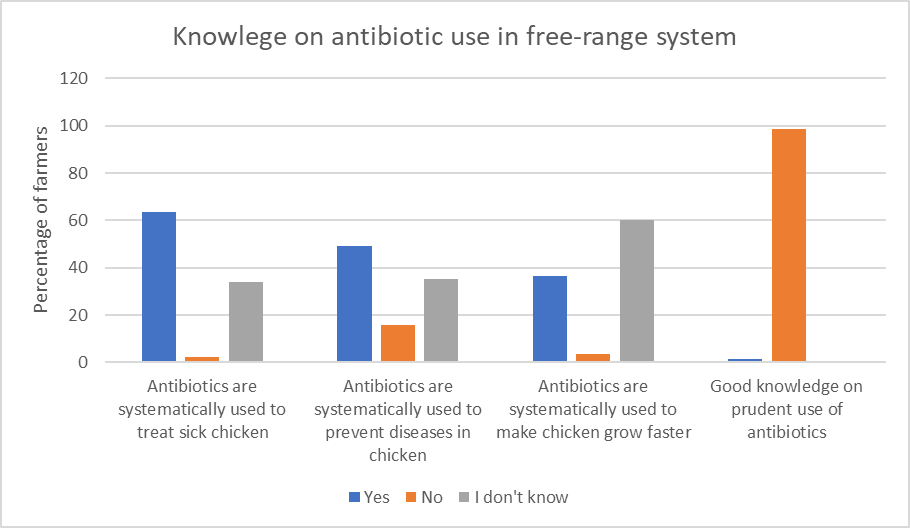


**Figure S1**. Level of knowledge on antibiotic use among farmers under free-range system in Uganda


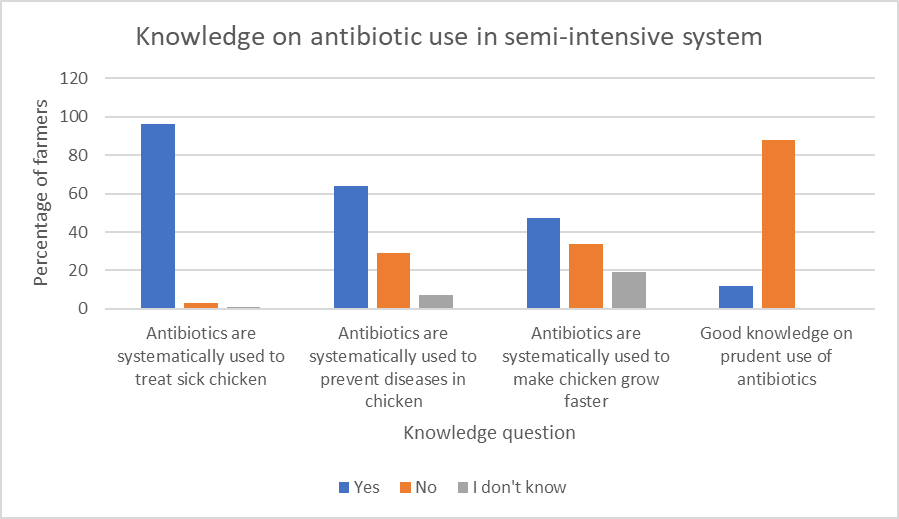


**Figure S2.** Level of knowledge of farmers on antibiotic use among farmers under semi-intensive system in Uganda

Supplementary Table S1: Modified AMUSE Questionnaire


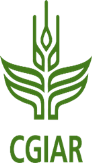

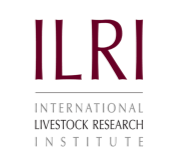

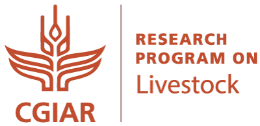

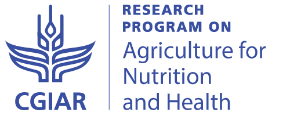


# AMUSE Livestock tool, version 2

**Criteria for selecting respondent:**

**person who plays a major role in the management of livestock**

| **Interview specifications** | | |
| --- | --- | --- |
| 1. Questionnaire ID | | *(Autogenerate and link to the sample)* |
| Name and contact | |  |
| 2. Date of Survey *(DD/MM/YYYY)* | | (Autogenerate) |
| 3. | Time interview started (HH:MM) | *(Automatically generated by tablet)* |
| 4. | Time interview ended (HH:MM) | *(Automatically generated by tablet)* |
| 5. | Interview done via interpreter | - Yes - No |

| **Enumerator specifications** |  |
| --- | --- |
| 6. Enumerator’s name *(First Name and Last Name)* |  |

| **Farm specifications** |  |
| --- | --- |
| 8. District/County | *(List of all districts pre-coded)/County* |
| 9. Sub-county | *(List of all sub counties pre-coded)* |
| 10. Parish/Ward | *(List of all parishes pre-coded)/Wards* |
| 11. Village/ Sub-location | *(List of all village pre-coded)/Sub- locations* |
| 12. GPS Coordinates | *(Automatically generated by tablet)* |

| **DEMOGRAPHICS** |  |
| --- | --- |
|  |  |
| 13. Respondent’s sex | - Male - Female |
| 14. What is the marital status? | - Single - Married - Divorced - Widowed? |
| 15. Are you the household head? | - Yes - No |
| 16. Who makes decisions about the following the farm business |  |
| 1. buying chicken | - Myself - My husband/wife - Other………………………………………………… |
| 1. purchasing drugs | - Myself - My husband/wife - Other………………………………………………… |
| 1. administering drugs to the poultry? | - Myself - My husband/wife - Other |
| 1. selling chicken/eggs | - Myself - My husband/wife - Other………………………………………………… |
| 17. Who decides on how the money from the sales should be used (chicken/eggs) | - Myself - My husband/wife - Other………………………………………………… |
| 18. What is your age group? | - 15-20 - 21-29 - 30-39 - 40-49 - 50-59 - 60-69 - >70 |

| 19. What is your education level? | - Primary school (P1-P7) (P8) - Secondary school (S1-S6) - Technical Institute - University |
| --- | --- |

| 20. Type of farm | - *Household farm* - *Commercial farm* |
| --- | --- |
| 21. How many chickens do you currently have? | - 0-50 - 51-200 - 201-500 - 500-1000 - >1000 |
| How would you characterize your production scale? (autogenerate based on qn.21) | - Traditional*1-50) - semi-intensive (51-2000) - intensive (>2000) |
| 22. What other avian species (other than chickens) are kept on the farm? | - None - Turkeys - Ducks - Guinea fowls - Others (specify)…………… |

| 23. What species of chicken are kept on the farm? | - Exotic chicken breed - Indigenous chicken breed - Cross bred/hybrid |
| --- | --- |
| 24. What is the main purpose of keeping birds | - layers (egg production) - mainly for own consumption - mainly for sale      - broilers (meat) - mainly for own consumption - mainly for sale      - produce Day Old Chicks (DOC ) - Dual purpose (meat and eggs) - Mainly for own consumption - Mainly for sale |

| 25. How are you keeping the chickens? | - Housed day and night - Free-range at day - housed at night - Free-range day and night |
| --- | --- |

| 26. What is the main source of your feeds? | - Purchase from Agrovet - Purchase from Feed companies - Household waste - Scavenging - Self-formulation |
| --- | --- |

| 28. Does the household use the chicken manure for any purpose? | - Yes - No | |
| --- | --- | --- |
| 1. If yes,   What do you do with the manure from the chickens? | - Use as fertilizer for own crops - Use as fuel (incl. biogas) - Sell it (or trade it) - Give it away - Leave it where dropped - other (specify)……. | |
| 29. What other animals do you have on your farm other than poultry?  *Let the respondent answer freely then probe for the other options* | - Cows/Cattle - Pigs - Goats - Sheep - Horses or donkeys - Camels - Dogs or cats | |
| 30. Is it easy for you to access Veterinary services (Veterinary doctor, drug shop, ) | | - Yes - No |
| 31. How far is the nearest vet drug shop from your farm | | - Less than 1 km - 2-5km - More than 5km |
| 32. Who is your primary provider of animal health service? | | - 1 traditional healer - 2 Community animal health worker - 3 Private veterinarian (qualification unknown) - 4 Private veterinarian (qualified) - 5 Official/government veterinarian - 6 other, specify…………………………………. |
| 33. Did you call for professional help, for example a qualified veterinarian in the last 4-8 weeks? | | - Yes - No |
| 34. Did you use laboratory services, for example for testing blood samples from your animals or submitting dead birds in the last 3-6months? | | - Yes - No |
| 35. | |  |
| 36. Have you reported any disease problems to any animal health service provider in the last 3 months? | | - Yes - No |
| Have you been visited by a drug/pharma company sales representative? | | - yes - No |

| 37. From the drugs shown on the chart, how many times have you used them in your poultry in the last 4 weeks. Alternatively ask to see packaging of commonly used drugs and classify according to list below.    For drugs used, please indicate the reason for use: multiple options for each drug is possible | | | | | | |
| --- | --- | --- | --- | --- | --- | --- |
| Drug | Number of times used in the last 4 weeks | Prevent disease | | Treat sick animal/Bird | Fattening/growth promotion | Other |
| 1. Vaccines |  |  | |  |  |  |
| 1. Anthelmintics |  |  | |  |  |  |
| 1. Ectoparasiticides |  |  | |  |  |  |
| 1. Tetracyclines |  |  | |  |  |  |
| 1. Sulphonamides |  |  | |  |  |  |
| 1. Penicillins (and combinations with   Clavulanic acid? or in combination with other antibiotics) |  |  | |  |  |  |
| 1. Fluoroquinolones |  |  | |  |  |  |
| 1. Macrolides |  |  | |  |  |  |
| 1. Aminoglycosides |  |  | |  |  |  |
| 1. Other antibiotics (specify) |  |  | |  |  |  |
| 1. Vitamins/Iron   supplements/mineral supplements |  |  | |  |  |  |
| 1. Other drugs (specify) |  |  | |  |  |  |
| 38. Do you use your own personal drugs (human) in your poultry? | | | - Yes - No | | | |
| 1. What is the reason for doing that? | | |  | | | |
| 39. Do you use animal drugs to treat yourself or a family member/friend? | | | - Yes - No | | | |
| 1. What is the reason for doing that? | | |  | | | |
| 40. Which period of the year do you think you use more drugs on the farm? | | | - December-February - March-May - June-August - September-November | | | |

| 41. For the two most commonly used drugs Q37 answer the following question (max 2 drugs)- Drug option 1 *Question 41 needs to be asked twice (for 2 drugs), add field for ‘drug’ ID, can be number of drug class above*  Name drug: | | |
| --- | --- | --- |
| a. Who administered the drug the last time you used it? | | - I myself or family member or farm worker - Veterinarian or animal health worker - Other: ___________________________ |
| b. Which chicken/birds were given the drug the last time you used it? | | - All birds - All birds of a certain age - Sick birds - Birds that should be sold - birds that were just bought |
| c. | Where did you get the drug from? *Enumerator to probe if veterinary drug store/Agrovet/ Human Pharmacy/from market for the specific name of the store to be used later while collecting veterinary drug samples* | - From veterinary drug store - From human pharmacy - From the vet - From other animal health service provider - From the market - From traders passing by - From friends/neighbours/family - Other:…………….. |
| d. | How did you know how to use the drug? | - My previous experience or knowledge - Advice from veterinarian - Advice from other animal health service provider   (e.g. community animal health worker)     - Advice from pharmacist/drug store/Agrovet - Advice from neighbour/friend - Advice from package/label of the drug - Advice from other: ________________ |
| e. | Was the drug successful? | - Yes, completely - Yes, partly - No |

| 42. For the two most commonly used drugs Q37 answer the following question (max 2 drugs)- Drug option 2 *Question 41 needs to be asked twice (for 2 drugs), add field for ‘drug’ ID, can be number of drug class above*  Name drug: | | |
| --- | --- | --- |
| a. Who administered the drug the last time you used it? | | - I myself - Veterinarian - Other: ___________________________ |
| b. Which chicken/birds were given the drug the last time you used it? | | - All birds - All birds of a certain age - Sick birds - birds that should be sold - birds that were just bought |
| c. Where did you get the drug from? *Enumerator to probe if veterinary drug store/Agrovet/ Human Pharmacy/from market for the specific name of the store to be used later while collecting veterinary drug samples* | | - From veterinary drug store - From human pharmacy - From the vet - From other animal health service provider - From the market - From traders passing by - From friends/neighbours/family 🞏 Other:……………….. |
| d. How did you know how to use the drug? | | - My own knowledge - Advice from veterinarian - Advice from other animal health service provider   (p.e. community animal health worker)     - Advice from pharmacist/drug store/Agrovet - Advice from neighbour/friend - Advice from package/label of the drug - Advice from other: ________________ |
| e. | Was the drug successful? | - Yes, completely - Yes, partly - No |
| f. | Where do you normally keep the drug (s) you bought? | - In a closed cabinet outside the chicken house - On an open shelf outside the chicken house - Inside the animal/chicken house - Used that same day - Other: ________________ |

| 43. How much did you spend on drugs for poultry in the last 4-8 weeks? Including all drugs mentioned in Q40 | | |
| --- | --- | --- |
| In total (in local currency) | |  |
| 1. Provide split of total if possible | |  |
| a. | Vitamins? | __________ 🞏 Don’t know |
| b. | Vaccines? | __________ 🞏 Don’t know |
| g. | Dewormer? | __________ 🞏 Don’t know |
| h. | Antibiotics? | __________ 🞏 Don’t know |
| i. | Acaricides | __________ 🞏 Don’t know |
|  | Any other drugs used during the last 3 months? | __________ |

| 44. What do you do to keep your poultry healthy, so they don’t get sick?  *Do not read options! Let them speak without telling them the options* | - Clean/disinfect - Use vet drugs (incl. vaccine) - Keep well fed - Special feed (incl. supplements) - Fencing - Avoid mixing with other herd/flock - Other: _____________________________ |
| --- | --- |

| 50. How many birds have died in the last 4 weeks? | - 0-10 - 10-100 - More than 100 |
| --- | --- |
| 51. When was the last time a chicken/bird was sick? | - <1 month ago - 1-6 months ago - 7-12 months ago - >12 months ago - Never been sick |
| 52. What kind of disease was it?  *(select most appropriate group based on clinical sign or disease name given)* | - Respiratory - Digestive/intestinal tract e.g. diarrhea - Reproductive - Sudden death - Skin disease/wounds - External parasites - Neurological signs - Other _____________________ |
| 53. What did you do when the chicken/birds were sick?  *(do not read option, select most suitable answer)* | - Use traditional medicine - Use medicine from the veterinary drug store (self-bought) - Consult traditional healer - Consults community animal health worker - Consult official veterinarian - Consult private veterinarian - Vet applied/left drugs - Other: _____________________________ |
| 54. Who did you turn to for help with diagnosis and treatment? | - No-one - Private veterinarian - Government veterinarian - Pharmacist/drug store - Other animal health service provider (p.e.   Community animal health worker)   - Neighbour/friend - Other: _____________________________ |
| 55. Do you keep records of treatments administered? | - 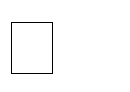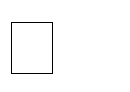Yes - No |
| 56. If yes, what kind of treatment records are kept? | - Paper - Electronic - Board - Other:………………………. |
| 57. What did you do with sick chicken/bird during and a few days after treatment? | - Used it normally (consume or sell eggs and/or chicken) - Isolated from rest of the flock - Threw it away |
| 58. What do you do if a sick chicken/ bird did not improve after treatment or died a few days after treatment? | - Used it normally (consume or sell) - Buried the dead animal - Burnt the dead animal - Threw it away - Fed to animals on the farm |

## Questions on farm

| 59. What type of work are you involved in when it comes to the farm? | - Managing the farm - Feeding and watering the birds - Other (specify) |
| --- | --- |
| 60. Do you disinfect the farm after every production cycle? | - Yes - No |
| 61.Do you have a foot bath? | - Yes - No |
| 1. If yes, do you add a disinfectant? | - Yes (specify) - No |
| 62. Are you involved in the daily work of feeding and taking care of the chickens? | - Yes - No |
| 63. Are you involved in selling of chickens or chicken products such as eggs? | - Yes - No |
| 64. Are you involved in treating or looking after the chicken when sick? | - Yes - No |

| 65. Do you have employees or casual workers that are involved in taking care of the chickens? | - Yes - No |
| --- | --- |

| 66. How long do you keep the birds on the farm | - 4 weeks or less - 4-8 weeks - 3-6months - 6 months- 12 months - Over a year |
| --- | --- |
| 67. Do you have egg laying birds on the farm? | - Yes - No |
| a. If yes,  a. Did you sell any eggs during the 4-8 weeks? | - Yes - No |
| 68. Do you sell eggs throughout the year, or at certain months or occasionally? | - Throughout the year - Certain months/periods - Occasionally |
| 69. Where did you sell the eggs? | - To Neighbours - To the market - To Middle men/ traders - To others |
| 70. Did you sell any live chicken during the last 3 months? | - Yes - No |
| 71. Did you sell live chicken throughout the year, at certain months or occasionally? | - Throughout the year - Certain months/periods - Occasionally |
| 72. Where did you sell the live chicken? | - To Neighbours - To the market - To Middle men/ traders - To others |
| 73.How are dead birds disposed of? | Buried  Composted  Fed to other animals  Other____ |
| 74.How often does the household consume eggs | - Everyday - Three times a week - Once a week - Once a month - Don’t consume |
| Where do you get the eggs, you consume? | - From the farm - Bought from outside |
| 75. Who in the household is given priority to consume eggs? | - The young - The old - Both young and old |
| 76. How many times does the household consume chicken meat? | - Everyday - Three times a week - Once a week - Once a month - Don’t consume |
| 77. Who in the household is given priority to consume chicken meat ? | - The young - The old - Both young and old |

| 78. Does your household get income (s) from the following? | | |
| --- | --- | --- |
| a. From selling vegetables or fruits? | | - Yes - No |
| b. From other livestock farming other than poultry? E.g.sale of milk or other meat | | - Yes - No |
| c. | Incomes from other business other than livestock and crop farming? E.g. brewing, mining, sale of forest products like wood or firewood, etc | - Yes - No - Other____________ |

| 79. How big a part of your entire household´s income comes from your poultry farming? | - 1 All of the income - 2 Major part of the income - 3 Half of the income - 4 Minor part of the income - 5 None of the income |
| --- | --- |

| 80. Would you say vaccination can be used for any of the following… | | |
| --- | --- | --- |
| a. To cure sick animals? | | - Yes - No - Don’t know |
| b. To prevent animals from being sick? | | 🞏 Yes   - No - Don’t know |
| c. | To make animals grow faster (fattening)? | - Yes - No - Don’t know |

| 81. Would you say antibiotics can be used for any of the following… | |
| --- | --- |
| a. To cure sick animals? | - Yes - No - Don’t know |
| b. To prevent animals from being sick? | - Yes - No - Don’t know |
| c. To make animals grow faster (fattening)? | - Yes - No - Don’t know |

|  |  |
| --- | --- |
| 82. Would you or your family members consume eggs from chicken/ birds that were recently treated with drugs including vaccinations? | - 1 Yes - 2 No |
| 83. If No to the above, how many days should you wait before consuming eggs from treated birds? | …………………days |
| 84. Would you consume meat from birds that were recently treated with drugs incl. vaccinations. | - 1 Yes - 2 No |
| 85. If No to the above, how many days should you wait before consuming the meat? | ……………………days |

| 86. If you hear two of your neighbors talk like this. Would you agree with neighbor A or neighbor B? | | |
| --- | --- | --- |
| a. A says: You should always dispose of expired drugs because they may turned bad  B says: Drugs are expensive and usually last longer than what the package says, so you can still use them | | - Agree with A - Agree with B - Don’t know |
| 87. What do you do with your expired drugs? | | 🞏 Continue to use   - Throw away drugs (e.g. in pit   Latrines, rubbish pit or bushes)   - Burn or bury - Give away to drug vendors to dispose for me - other, please specifiy……….. |
|  | 88. Have you ever attended any training for farmers on disease prevention and control in the last 24 months (2 years)? | - Yes - No |
|  | 89. What were you trained on? Multiple answers possible | - Detecting animal diseases ] - Reporting animal diseases - Treating animal diseases - Use of veterinary drugs etc - Other   - |
|  | 90. Who provided the trainings? (max 3 answers) | - Government /official vets - Private vets - Extension system - NGOs - Church organization - Farmer Cooperatives - Drug company - Pharmacy/ Agrovet owners - Others |
|  | 91. What do you remember from the training? *Open question* |  |
|  | 92. Did you change your farming? |  |
|  | If yes, what did you change after the training? *Open question* |  |
